# Recent Advances in Bioactive Compounds, Health Functions, and Safety Concerns of Onion (*Allium cepa* L.)

**DOI:** 10.3389/fnut.2021.669805

**Published:** 2021-07-22

**Authors:** Xin-Xin Zhao, Fang-Jun Lin, Hang Li, Hua-Bin Li, Ding-Tao Wu, Fang Geng, Wei Ma, Yu Wang, Bao-He Miao, Ren-You Gan

**Affiliations:** ^1^Institute of Urban Agriculture, Chinese Academy of Agricultural Sciences, Chengdu, China; ^2^Chengdu National Agricultural Science and Technology Center, Chengdu, China; ^3^Burnett School of Biomedical Sciences, University of Central Florida, Orlando, FL, United States; ^4^Guangdong Provincial Key Laboratory of Food, Nutrition, and Health, Department of Nutrition, School of Public Health, Sun Yat-sen University, Guangzhou, China; ^5^Key Laboratory of Coarse Cereal Processing (Ministry of Agriculture and Rural Affairs), Sichuan Engineering and Technology Research Center of Coarse Cereal Industralization, Chengdu University, Chengdu, China

**Keywords:** phytochemicals, antioxidant, anticancer, anti-obesity, anti-diabetic, safety

## Abstract

Onion (*Allium cepa* L.) is a common vegetable, widely consumed all over the world. Onion contains diverse phytochemicals, including organosulfur compounds, phenolic compounds, polysaccharides, and saponins. The phenolic and sulfur-containing compounds, including onionin A, cysteine sulfoxides, quercetin, and quercetin glucosides, are the major bioactive constituents of onion. Accumulated studies have revealed that onion and its bioactive compounds possess various health functions, such as antioxidant, antimicrobial, anti-inflammatory, anti-obesity, anti-diabetic, anticancer, cardiovascular protective, neuroprotective, hepatorenal protective, respiratory protective, digestive system protective, reproductive protective, and immunomodulatory properties. Herein, the main bioactive compounds in onion are summarized, followed by intensively discussing its major health functions as well as relevant molecular mechanisms. Moreover, the potential safety concerns about onion contamination and the ways to mitigate these issues are also discussed. We hope that this paper can attract broader attention to onion and its bioactive compounds, which are promising ingredients in the development of functional foods and nutraceuticals for preventing and managing certain chronic diseases.

## Introduction

Onion (*Allium cepa* L.) is widely cultivated and consumed around the world (1). The common onion varieties with three different colors, including red, yellow, and white, are normally available in the food market. As a food item, onion is usually served as a vegetable ingredient in warm dishes by cooking, like baking, boiling, braising, grilling, frying, roasting, sautéing, or steaming. It can also be eaten raw in salads, made into juice, pickled in vinegar, or used as a spice. As an herbal medicine, onion is recommended to relieve or prevent several common diseases, such as atherosclerosis, asthma, bronchitis, and coughs. The health benefits of onion are mainly attributed to its diverse bioactive constituents, such as organosulfur compounds, phenolic compounds, polysaccharides, and saponins (2, 3). Recently, accumulated studies demonstrated the remarkable health functions of onion and its bioactive compounds, including antioxidant (4), antimicrobial (5), anti-inflammatory (6), anti-obesity (7), anti-diabetic (8), anticancer (9), cardiovascular protective (10), neuroprotective (11), hepatorenal protective (12), respiratory protective (13), digestive system protective (14), reproductive protective (15), and immunomodulatory properties (16). Generally speaking, onion consumption is quite safe for the consumers. However, several potential health concerns should not be ignored, such as pesticide residue (17), heavy metal-enrichment (18, 19), microbial contamination (20, 21), and nitrate accumulation (22).

**Figure 1:**
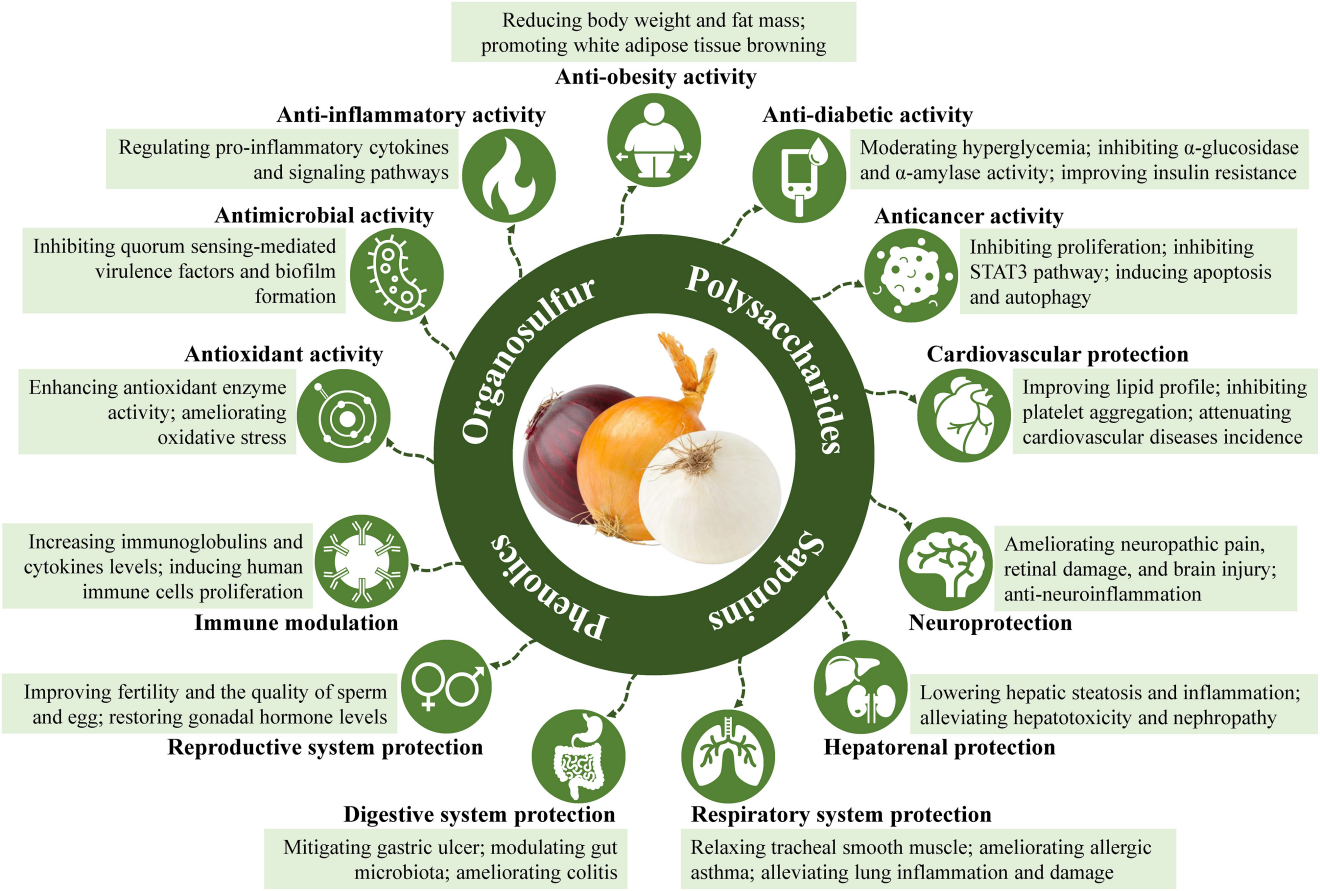
Bioactive compounds and health functions of onion.

Although the bioactive compounds and certain bioactivities of onion have been discussed in recent reviews (3, 23, 24), this review can provide an updated and more comprehensive understanding about the diverse health functions and safety concerns of onion. The literature summarized in this review was mainly collected from Web of Science Core Collection, PubMed, and Scopus databases from 2016 to 2021, with a focus on the bioactive compounds and health functions of onion, with special attention paid to the relevant molecular mechanisms (Figure 1). The potential safety concerns of onion and the strategies to mitigate these health risks are also discussed. It is expected to attract more attention to the health benefits of onion and its consumption and application in the prevention and management of chronic diseases.

## Bioactive Compounds in Onion

**Figure 2:**
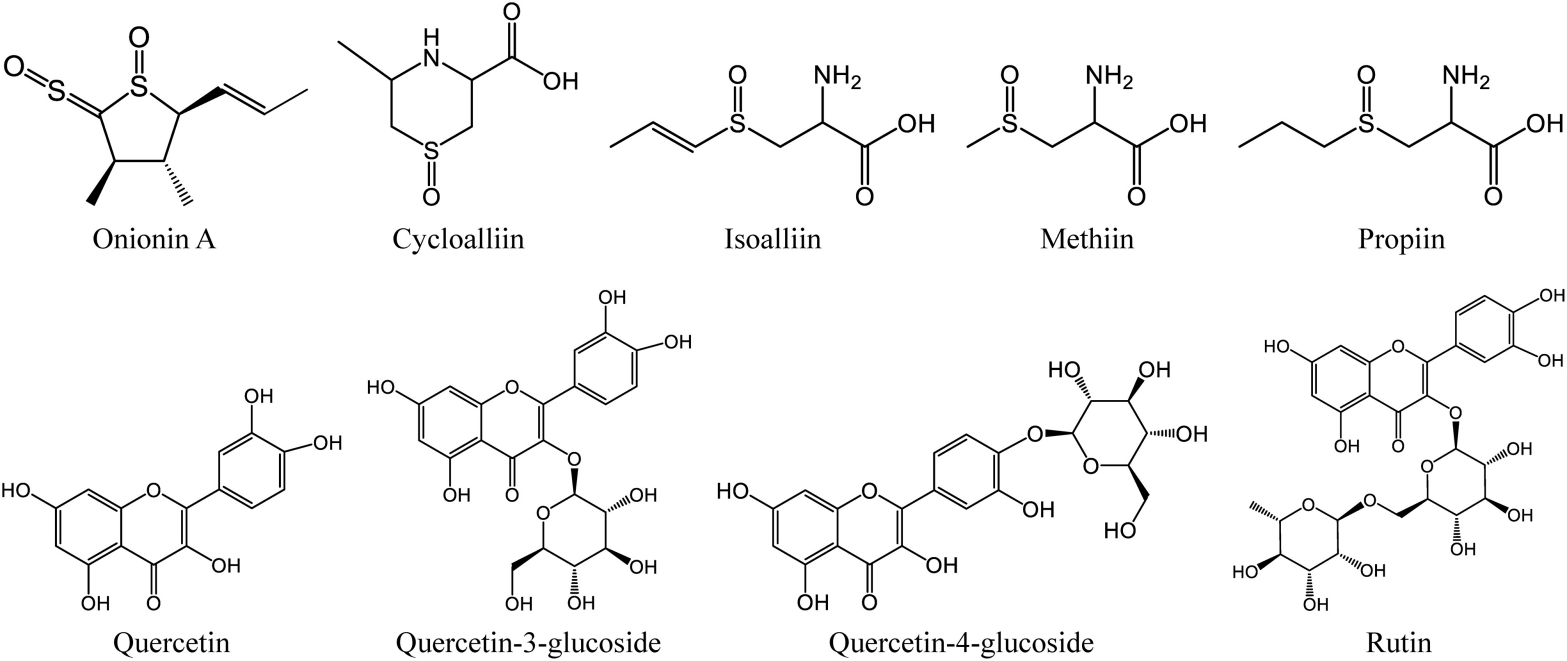
The chemical structures of the main organosulfur and phenolic compounds in onion.

Onion is rich in a variety of phytochemicals with beneficial functional, including organosulfur compounds (25, 26), phenolic compounds (27–29), polysaccharides (30), and saponins (31, 32). The major bioactive compounds of onion are sulfur-containing compounds, such as onionin A and cysteine sulfoxides, as well as the phenolic compounds, such as rutin, quercetin, and quercetin glucosides (Figure 2). It is different for the contents of bioactive compounds among different onion varieties (5). Red onion had the highest contents of anthocyanins and flavonols, followed by the yellow onion, but the white onion contained the lowest amount (33). Besides, the major compounds varied in different layers of onion (34). Quercetin was the major compound in the skin of red onion, while quercetin-4-glucoside was the main compound in its bulb (35).

Processing can change the bioaccessibility and content of bioactive compounds in onion. The bioaccessibility of total flavonols in onion was not affected by high-pressure processing, but the onion matrix could improve the bioaccessibility of its flavonol (36). It has been found that onion skin quercetin aglycone was more bioavailable than pure quercetin dihydrate in humans (37). The quercetin content was not significantly changed by sautéing (38), but the content and bioaccessibility of phenolic compounds, especially quercetin-derivatives, were found increased by cooking, such as baking, grilling, and frying (39). Besides, the contents of the cysteine sulfoxides, including cycloalliin, isoalliin, methiin, and propiin, were changed differently in onion by heat processing, depending on the cooking methods (40). For instance, their contents were decreased during boiling, but increased during frying, microwaving, and steaming. Furthermore, the flavonoid content was decreased during the processing of black onion, while the contents of isoalliin and fructose were significantly increased (41).

## Health Functions of Onion

Many plant-based foods, such as garlic (42), ginger (43), sweet tea (44), dark tea (45), germinated edible seeds and sprouts (46), as well as their bioactive compounds, including resveratrol (47), curcumin (48), rutin (49), quercetin (50), citrus flavonoids (51), and spice essential oils (52) have been demonstrated to possess a variety of health functions. As a traditional and popular food, onion has also been reported with multiple health functions based on *in vitro, in vivo*, and human studies (Table 1). In the following part, the main health functions and related molecular mechanisms of onion and its bioactive compounds are summarized and discussed in detail.

**Table 1:** The health functions and potential mechanisms of onion.

**Product/compounds**	**Study type**	**Subjects/cell lines**	**Dose**	**Main effects and potential mechanisms**	**References**
**Antioxidant activity**					
Onion methanol extract	*In vitro*	Rat dopaminergic cell line N27-A	500 μg/mL	Upregulating antioxidant enzyme (HO-1, NQO1, and catalase) expressions	(6)
Onion powder	*In vivo*	Rats	10% onion powder in diets	Enhancing the activity of CAT, SOD and GPx enzymes in erythrocytes and liver	(10)
Onion extract	*In vivo*	Ovalbumin-sensitized rats	35, 70, and 140 mg/kg/d for 21 days	Enhancing the levels of SOD, CAT, and thiol	(53)
Onion	*In vivo*	Potassium bromate-induced oxidative damage in rats	10, 20, and 30% in diets	Protecting against oxidative damage; reducing MDA levels; restoring the architecture of liver and kidney cells	(54)
Phenolic-rich onion extract	*In vivo*	Broiler chicks	1, 2, and 3 g/kg diet	Increasing CAT, SOD activity, and GSH level	(55)
Pawpaw-onion powder	*In vivo*	African catfish	2.5, 5, and 10 g/kg diet	Increasing antioxidant enzyme activity	(56)
Onion juice	Clinical trial	Healthy subjects	100 mL for 8 weeks	Reducing total free radicals and superoxide anions levels; elevating the glutathione content and total antioxidant capacity	(57)
**Antimicrobial activity**					
Onion liquid and lyophilized extracts	*In vitro*	*Staphylococcus aureus; Escherichia coli*		Showing high antibacterial efficiency against Gram-positive bacteria, such as *S. aureus*	(58)
Lemongrass and onion essential oils	*In vitro*	*Escherichia coli; Salmonella* Choleraesuis*; Listeria monocytogenes; Staphylococcus aureus*		Leafy vegetables treated with the essential oils combination showed higher antibacterial protection and odor acceptability	(59)
Red onion polysaccharide fractions	*In vitro*	*Staphylococcus aureus; Escherichia coli; Bacillus subtilis; Salmonella typhimurium*		Showing stronger antibacterial effect against *B. subtilis* than other bacteria	(30)
Graphene using extract of onion	*In vitro*	*Escherichia Coli; Pseudomonas aeruginosa; Staphylococcus faecalis; Staphylococcus aureus*		Showing great antibacterial activity	(60)
Bulb extracts from onion and onion varieties	*In vitro*	*Bacillus cereus; Staphylococcus aureus; Listeria innocua; Escherichia coli; Pseudomonas aeruginosa*		Against three Gram-positive species (*B. cereus, L. innocua, S. aureus*) and *P. aeruginosa*	(5)
Onion husks non-polar fraction; 7-Keto-(5-6-dihydro)-β-Sitosterol	*In vitro*	*Pseudomonas aeruginosa; Chromobacterium violaceum*		Inhibiting Quorum sensing effects; inhibiting swimming motilities	(61)
Silver nanoparticles using extracts of neem, onion and tomato	*In vitro*	*Staphylococcus aureus*		Against Gram-positive bacteria *Staphylococcus aureus* in nutrient agar	(62)
Red onion skin extract	*In vitro*	*Staphylococcus epidermidis; Staphylococcus aureus; Listeria innocua; Enterococcus faecalis*		Showing great antibacterial activity	(63)
Onion essential oil	*In vitro*	*Aspergillus, Fusarium*, and *Penicillium* species		Showing fungicidal or inhibitory effects on the growth of fungal species from food	(64)
Onion endophytic bacterium *Bacillus endophyticus*	*In vitro*	*Magnaporthe oryzae*		Showing effective antifungal effect against rice blast pathogen	(65)
Red onion ethanol extract	*In vitro*	*Trichophyton rubrum*		Preventing tinea pedis caused by fungal infection	(66)
**Anti-inflammatory activity**					
Onion methanol extract	*In vitro*	Lipopolysaccharide-induced BV-2 microglial cells	50, 250, and 500 μg/mL	Preventing LPS-stimulated increases of proinflammatory cytokines, TNF-α, IL-6, and IL-1β; decreasing iNOS and COX-2 levels; reducing the release of NO	(6)
Red onion skin extract	*In vitro*	LPS-treated RAW 264.7 cells		Inhibiting IL-6 and IL-1; decreasing the release of NO	(63)
Onion-derived nanoparticles	*In vitro*	LPS-treated RAW 264 cells		Inhibiting NO production	(67)
Onion bulb extract	*In vitro*	Isolated bone-marrow derived neutrophils	0.01, 0.1, 1, 10, and 100 μg/ml	Reversing and preventing colitis in mice *via* inhibition of proinflammatory signaling molecules and neutrophil activity	(14)
	*In vivo*	Dextran sulfate sodium-induced colitis in mice	100 and 200 mg/kg		
Onion extract	*In vivo*	Rats	0.175, 0.35, and 0.7 mg/mL in drinking water	Decreasing in total WBC and PLA2 level; decreasing neutrophil and eosinophil counts; increasing in lymphocytes count	(68)
Onion bulb extract	*In vivo*	Dextran sulfate sodium-induced colitis in mice	30, 60, 100, and 200 mg/kg	Modulating the expression and the activity of important pro-inflammatory molecules and signaling pathways involved in the inflammatory response	(69)
Onion bulb extract	*In vivo*	Mice	10, 30, 60, and 100 mg/kg	Alleviating house dust mite-induced perivascular and peribronchial inflammation through EGFR, ERK1/2, and AKT pathway	(13)
Onion aqueous extract	*In vivo*	Carrageenan-induced paw edema in rats	0.1, 0.5, and 1.5 mg/kg I.P injection	Reducing rat paw edema dose dependently	(70)
**Anti-obesity activity**					
Onion peel extracts; quercetin and isoquercetin	*In vitro*	3T3-L1 cells	Extract 50, 100, and 150 μg/ml; quercetin and isoquercetin 25, 50, and 100 μM	Remodeling white adipocytes to brown-like adipocytes	(27)
	*In vivo*	HFD-fed mice	0.5% in diets for 8 weeks		
Onion peel extract	*In vitro*	3T3-L1 cells	25, 50, 100, 150, 200, 300, 400, and 500 μg/mL	Inhibiting lipid accumulation	(71)
	*In vivo*	HFD-fed mice	36, 90, and 144 mg/kg for 8 weeks	Reducing body weight; lowering fat coefficient and improving serum lipid levels	
Quercetin; red onion extract	*In vivo*	HFD-fed mice	Diets with 17 mg/kg of quercetin equivalents for 9 weeks	Preventing hypermethylation in the Pgc-1α promoter	(72)
Onion oil	*In vivo*	HFD-fed rats	46.3 and 92.6 mg/kg/d for 60 days	Reducing body weight gain and tending to decrease adipose tissue weight	(73)
Quercetin-rich onion peel extract	Clinical trial	72 subjects with BMI > 23 kg/m^2^	170 mg capsule contains 50 mg quercetin, 2 capsules/d for 12 weeks	Reducing weight and percentage of body fat; decreasing blood glucose and leptin levels	(7)
Quercetin-rich onion powder	Clinical trial	70 healthy Japanese subjects	9 g/d for 12 weeks	Lowering alanine aminotransferase; reducing visceral fat area in lower high-density lipoprotein cholesterol subjects	(74)
Steamed onion	Clinical trial	70 overweight subjects	300 mg capsule contains 37.5% steamed onion; 3 capsules/d for 12 weeks	Reducing percentage of body fat and fat mass with no significant effects on lean body mass	(75)
Onion peel extract	Clinical trial	61 overweight and obese subjects	Capsule contains 50 mg quercetin, 119.2 mg total polyphenol, and 65.0 mg total flavonoid; 2 capsules/d for 12 weeks	Regulating erythrocyte n-6/n-3 ratio and preventing fat accumulation in various body regions	(76)
**Anti-diabetic activity**					
Onion seed extract	*In vivo*	Streptozotocin-induced male rats	200 and 400 mg/kg/d for 28 days	Protecting against adverse effects of diabetes on reproductive system	(77)
Fenugreek seeds and onion	*In vivo*	Streptozotocin-induced diabetes in rats	10% fenugreek seeds or 3% onion or their combination in diet for 6 weeks	Ameliorating hyperglycemia and its associated metabolic disorders	(78)
Fenugreek seeds and onion	*In vivo*	Streptozotocin-induced diabetes in rats	10% fenugreek seeds or 3% onion or their combination in diet for 6 weeks	Reducing oxidative stress	(79)
Fenugreek seeds and onion	*In vivo*	Streptozotocin-induced diabetes in rats	10% fenugreek seeds or 3% onion or their combination in diet for 6 weeks	Alleviating cardiac damage	(80)
Fenugreek seeds and onion	*In vivo*	Streptozotocin-induced diabetes in rats	10% fenugreek seeds or 3% onion or their combination in diet for 6 weeks	Attenuating diabetic nephropathy	(81)
Fenugreek seeds and onion	*In vivo*	Streptozotocin-induced diabetes in rats	10% fenugreek seeds or 3% onion or their combination in diet for 6 weeks	Ameliorating eye lens abnormalities	(82)
Fenugreek seeds and onion	*In vivo*	Streptozotocin-induced diabetes in rats	10% fenugreek seeds or 3% onion or their combination in diet for 6 weeks	Countering the deformity and fragility of erythrocytes	(83)
Red onion scales extract	*In vivo*	Streptozotocin-induced diabetes in rats	150 and 300 mg/kg/d for 4 weeks	Improving fasting blood glucose and advanced glycation end products levels; elevating serum insulin level; down-regulating inflammatory mRNA expression	(84)
Heat-processed onion extract	*In vivo*	Male rats	500 mg/kg	Showing anti-diabetic effect by suppressing carbohydrate absorption *via* inhibition of intestinal sucrase, thereby reducing the post-prandial increase of blood glucose	(85)
Fenugreek seeds and onion	*In vivo*	Streptozotocin-induced diabetes in rats	10% fenugreek seeds or 3% onion or their combination in diet for 6 weeks	Attenuating diabetic nephropathy *via* suppression of glucose transporters and renin-angiotensin system	(86)
Onion peel extract/onion powder	*In vivo*	Alloxan-induced diabetes in rats	1 and 3% onion peel extract, 5 and 7% onion powder in bread	Reducing blood glucose and MDA levels; improving antioxidant enzyme activities	(87)
Raw red onion	clinical trial	53 overweight or obese non-diabetic patients with polycystic ovary syndrome	2 × 40–50 g/d for overweight and 2 × 50–60 g/d for obese patients or 2 × 10–15 g/d for 8 weeks	Improving insulin resistance markers; increasing the chance of menses occurrence	(88)
**Anticancer activity**					
Onion methanolic extract	*In vitro*	MDA-MB-231 cells; A1235 cells	100 μg/mL in a complete medium	Inhibiting tumor cells proliferation	(89)
Onion varieties extract	*In vitro*	Caco-2 cells	1:10 dilution (10 μL of extract with 90 μL of growth media)	Inhibiting tumor cells proliferation	(90)
Onion bulb extract	*In vitro*	HeLa cells; HCT116 cells; U2OS cells	IC_50_: 24.79 μg/mL for HeLa; 24.73 μg/mL for HCT116; 36.6 μg/mL for U2OS	Inducing apoptosis in cancer cells	(91)
Flavonol glucosides from red onion waste	*In vitro*	HeLa cells	5, 10, 20, 50, and 100 μM	QG, QDG, isoquercetin, and spiraeoside showed potent anticancer effect	(92)
Spiraeoside from red onion skin	*In vitro*	HeLa cells	0.1, 1, 10, 50, and 100 ug/mL	Inhibiting cell growth; promoting apoptosis by activating caspase-3 and caspase-9; inhibiting the expression of cyclin-dependent kinase 2-cyclin-E	(93)
Onion extract	*In vitro*	AsPC-1, MCF-7, HCT116, HEP2, and HepG2	Encapsulated on nano chitosan	Decreasing IC_50_ in cancer cell lines; inducing apoptosis by down-regulating BCL-2 level and up-regulating the activity of caspase-3 and caspase-9	(94)
Onionin A from onions	*In vitro*	LM8 cells	4 and 20 mg/kg	Inhibiting tumor proliferation by suppressing Stat3 activation; inhibiting subcutaneous tumor development and lung metastasis	(95)
	*In vivo*	LM8 murine tumor-implanted model	20 mg/kg		
Fresh yellow onion	Clinical trial	Breast cancer patients during doxorubicin-based chemotherapy	30–40 g/d and 100–160 g/d for 8 weeks	Ameliorating hyperglycemia and insulin resistance during doxorubicin-based chemotherapy	(96)
**Cardiovascular protection**					
Onion methanol fractions and flavonols	*In vitro*	Collagen-induced platelet aggregation in rat platelet-rich plasma	0.5, 1, 3, and 5 mg/mL onion methanol fractions; 0.5, 1, and 2 mg/mL quercetin glycosides	Inhibiting platelet aggregation	(97)
Onion peel extract	*In vivo*	High-cholesterol diet-induced male mice	100 and 200 mg/kg/d for 12 weeks	Lowering liver weight, total cholesterol, LDL cholesterol, triacylglycerol, atherogenic index, and cardiac risk factor; increasing fecal cholesterol levels	(98)
Onion bulb powder	*In vivo*	High-cholesterol diet-induced male rats	10% onion powder in high-cholesterol diets for 7 weeks	Altering fecal bile acid composition by modulating microbiome	(99)
Onion bulb powder	*In vivo*	High-cholesterol diet-induced male rats	10% onion powder in high-cholesterol diets for 7 weeks	Modulating hepatic prostaglandins; enhancing ω-3 oxylipins in the liver; modifying sphingolipids in liver and spleen tissue	(100)
Onion bulb powder	*In vivo*	High-cholesterol diet-induced male rats	10% onion powder in high-cholesterol diets for 7 weeks	Increasing SOD, CAT, and GPx activities; anti-inflammatory response, and cardiovascular risk biomarkers	(10)
Onion extract	*In vivo*	High-cholesterol diet-induced male rats	0.5, 1.5, and 4.5 g/kg/d for 4 weeks	Alleviating hyperlipidemia with downregulation of HMGCR and upregulation of LDL receptor	(101)
Red wine extract of onion	Clinical trial	Healthy hypercholesterolemic volunteers	250 mL/d (contains 10.5% of alcohol, 1.4 g/L polyphenols, and 170 mg/L total flavonoids) for 10 weeks	Altering cholesterol; improving antioxidation; inhibiting inflammatory marker levels; attenuating cardiovascular disease incidence	(102)
Quercetin from onion skin extract powder	Clinical trial	Overweight to obese adults with hypertension	Capsule contains 132 mg onion skin extract powder, eq. 54 mg quercetin	Acute intake of quercetin does not influence post-prandial blood pressure and endothelial function	(103)
Quercetin from onion skin extract powder	Clinical trial	Overweight to obese patients with (pre-) hypertension	3 capsules/d (eq. 162 mg quercetin) for 6 weeks	Quercetin did not affect glucose, insulin, blood biomarkers of liver and renal function, hematology, and serum electrolytes	(104)
**Neuroprotection**					
Onion methanol extract	*In vitro*	Rat dopaminergic cell line N27-A	500 μg/mL	Upregulating antiapoptotic gene (Bcl-2); protecting against MPP^+^-induced death	(6)
Red onion ethanolic extract	*In vivo*	Streptozotocin-induced rats	125 and 250 mg/kg/d for 4 weeks	Improving learning and memory impairments in diabetic rats	(105)
Onion leave extract	*In vivo*	Rats with neuropathic pain	25, 50, and 100 mg/kg	Ameliorating diabetes-induced and chronic constriction injury-induced neuropathic pain	(106)
Onion ethanolic extract	*In vivo*	6-hydroxydopamine-induced rats	50, 100, and 200 mg/kg/d	Reducing malondialdehyde levels; ameliorating cognitive dysfunction	(11)
Onion water extract	*In vivo*	Pterygopalatine artery ligated mice	300 mg/kg	Ameliorating retinal damage by regulating the expression of neurotrophic factors	(107)
Onion outer scale extract	*In vivo*	Mice with cerebral ischemia-reperfusion injury	Ethyl acetate fraction: 85 and 170 mg/kg; aqueous fraction: 115 and 230 mg/kg	Improving the memory and sensorimotor functions in cerebral injury	(108)
**Hepatorenal protection**					
Onion juice	*In vivo*	Doxorubicin-induced rats	1 mL for 14 days	Preventing doxorubicin -induced hepatotoxicity	(109)
Red onion peel extract	*In vivo*	CCl_4_-induced rat hepatorenal toxicity	50 and 100 mg/kg	Ameliorating hepatonephro-linked serum and tissue markers dose dependently	(12)
Onion bulb powder	*In vivo*	High-cholesterol diet-induced rats	10% in diets for 7 weeks	Increasing liver SOD and GPx activity; decreasing liver protein carbonyls	(10)
Red onion scales extract	*In vivo*	Streptozotocin-induced rats	150 and 300 mg/kg/d for 4 weeks	Ameliorating kidney histopathological alterations	(84)
Onion powder	*In vivo*	High-fat, high sugar diet rats	7% in diets for 7 weeks	Lowering hepatic steatosis and hepatic TNF-α gene expression	(110)
Quercetin-rich onion powder	Clinical trial	Healthy Japanese subjects	9 g/d for 12 weeks	Improving liver function; lowering alanine aminotransferase level	(74)
**Respiratory system protection**					
Onion extract	*In vitro*	Isolated rat tracheal smooth muscle	2, 4, 8, 16, 32, and 64 mg/ml add to organ bath every 5 min	Relaxing tracheal smooth muscle *via* calcium channel blockade or β2-adrenergic stimulatory	(111)
Onion aqueous-alcoholic extract	*In vivo*	Asthmatic rats sensitized with ovalbumin	0.175, 0.35, and 0.7 mg/mL in drinking water	Decreasing tracheal responsiveness, neutrophil and eosinophil counts; increasing lymphocytes count; reducing monocyte count	(68)
Onion extract	*In vivo*	Nicotine-induced lung damage in rats	50 mg/kg/d	Attenuating the pathological effect of nicotine in the lung	(112)
Onion bulb extract	*In vivo*	House dust mite-challenged male mice	10, 30, 60, and 100 mg/kg	Mediating anti-inflammatory effects through the inhibition of the EGFR/ERK1/2/AKT-dependent pathway	(13)
**Digestive system protection**					
Red onion suspension	*In vivo*	Rats	200 and 500 mg/kg	Mitigating various experimental triggers of gastric mucosal injury	(113)
Onion powder	*In vivo*	Broiler chicks	1.5, 2, and 2.5 g/kg in diet	Improving the population of gut microflora and intestinal histomorphology	(114)
Onion quercetin monoglycosides	*In vivo*	High-fat diet fed rats	0.15% (quercetin:quercetin monoglycosides, 98:2 and 69:31) in diet for 4 weeks	Increasing the enzymatic activity of the intestinal microbiota	(115)
Onion quercetin monoglycosides	*In vivo*	Dextran sulfate sodium-induced colitis in mice	0.15% (quercetin:quercetin monoglycosides, 98:2 and 69:31) in diet	Reducing dextran sulfate sodium-induced colitis	(116)
Onion bulb extract	*In vivo*	Dextran sulfate sodium-induced colitis in mice	30, 60, 100, and 200 mg/kg	Reducing colitis severity; regulating expression and activity of pro-inflammatory molecules and signaling pathways	(69)
Onion bulb extract	*In vitro*	Isolated bone-marrow derived neutrophils	0.01, 0.1, 1, 10, and 100 μg/ml	Reducing the percentage of viable bone-marrow derived neutrophils; increasing spontaneous apoptosis	(14)
	*In vivo*	Dextran sulfate sodium-induced colitis in mice	100 and 200 mg/kg	Reducing colitis severity; regulating colonic expression/activity profile of pro-inflammatory molecules	
Phenolic-rich onion extract	*In vivo*	Broiler chicken	1, 2, and 3 g/kg in diet	Improving growth rate by improving amino acid ileal digestibility and intestinal histology	(55)
**Reproductive system protection**					
Cysteine sulfoxides	*In vitro*	Testis-derived I-10 cells	0.3, 1, and 3 mg/mL	Enhancing progesterone production *via* activation of the protein kinase A pathway	(117)
Onion juice	*In vivo*	Rats	3 mL/d	Increasing testosterone level	(118)
Onion juice	*In vivo*	Rats	3 mL/d	Against permethrin-induced testis damages	(119)
Onion juice	*In vivo*	Rats	3 mL/d	Maintaining reproductive ability and improving sexual activities	(120)
Onion juice	*In vivo*	Rats	3 mL/d	Restoring permethrin-induced reductions in hormonal of FSH and LH levels, and gene expression of LHCGR and SF1	(121)
Onion juice	*In vivo*	Rats	40 mg/kg/d	Improving the sperm quality and fertility after testicular torsion/detorsion	(15)
Onion extract	*In vivo*	Rats	100 and 1,000 mg/kg	Improving sperm count, motility, and morphology; ameliorating sera testosterone and SOD levels	(122)
Onion extract	*In vivo*	Rats	500 mg/kg	Protecting against dexamethasone-induced testicular damage in rats	(123)
Onion juice	*In vivo*	Rats	5 mL/kg for 21 days	Protecting against maternal dexamethasone-induced reproductive toxicity in rat female offspring	(124)
Onion juice	*In vivo*	Rats	Intracavernosal injection of 200 uL	Improving dutasteride-induced erectile dysfunction in rats	(125)
Onion extract	*In vivo*	Brown laying hens	0.0032% in diet	Improving egg quality and productive performance	(126)
**Immune modulation**					
Onion bulb extract	*In vitro*	NK CD^16+^ immune cells		Inducing the growth of CD^16+^ natural killer cells	(127)
Onionin A	*In vitro*	CD^4+^ and CD^8+^ cells	10, 30, 50, and 100 μM	Improving the activity of lymphocytes	(95)
	*In vivo*	Tumor-bearing mice	20 mg/kg/d for 2 weeks	Preventing the immunosuppressive activities of macrophages	
Onion extract	*In vivo*	Immune-suppressed rats	500 mg/kg/d for 4 weeks	Increasing the levels of cytokines (TNF and IL-6) and immunoglobulins (IgG and IgM)	(16)
Onion extract	*In vivo*	Ovalbumin-sensitized rats	35, 70, and 140 mg/kg/d for 21 days	Decreasing the levels of IL-4 and IgE	(53)

### Antioxidant Activity

Onion is a good source of natural antioxidants (128). Many studies have been carried out to evaluate the antioxidant activities of onion, and found that onion exhibits strong antioxidant properties by using a series of *in vitro* assays, including 2,2′-azino-bis-(3-ethylbenzothiazoline-6-sulfonic acid) (ABTS), 1,1-diphenyl-2-picrilhydrazyl (DPPH), ferric reducing antioxidant power (FRAP), lipid peroxidation, oxygen radical absorbance capacity (ORAC), total antioxidant capacity (TAC), and trolox equivalent antioxidant capacity (TEAC) assays.

**Figure 3:**
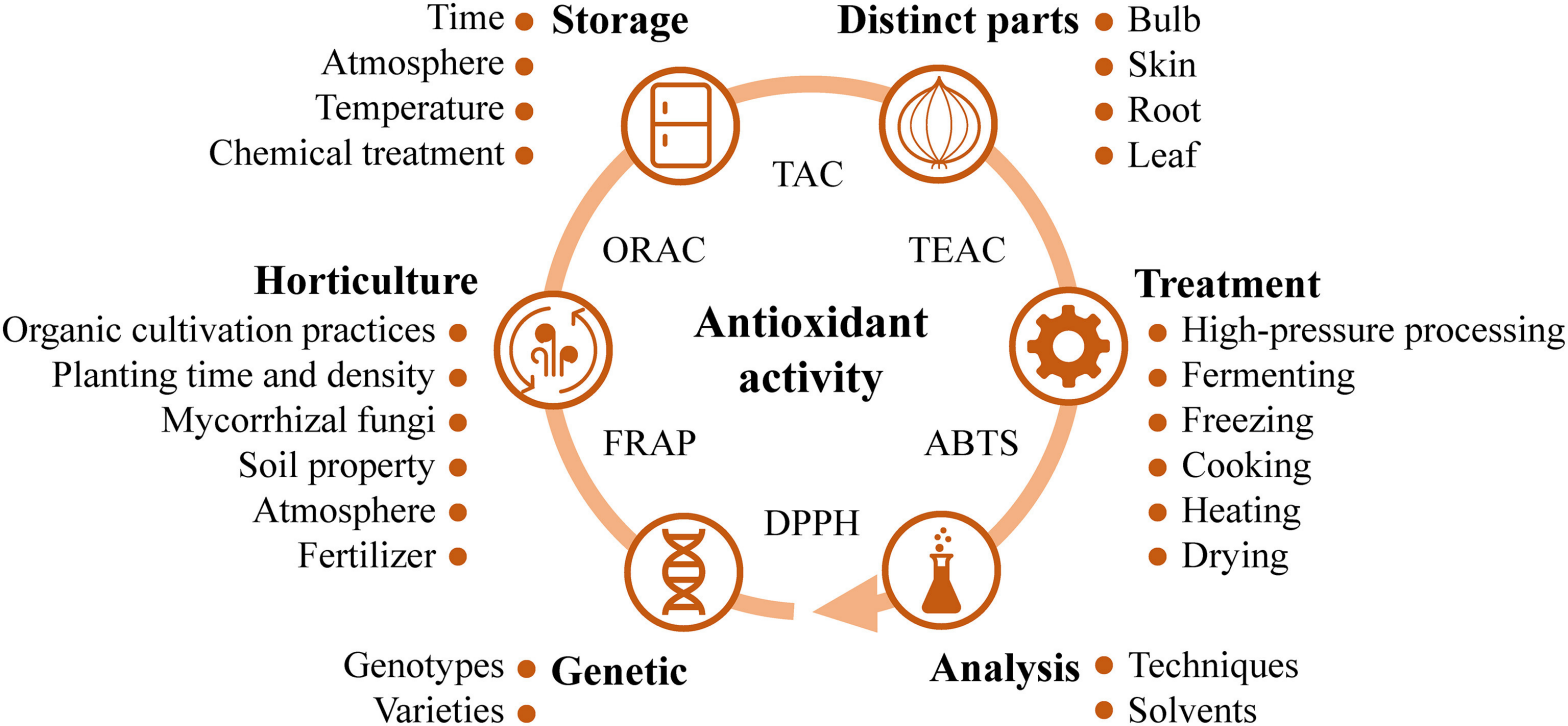
The main influence factors for antioxidant activity of onion.

Many factors were reported to influence the antioxidant activity of onion, such as the genetic background, horticultural techniques, storage conditions, distinct parts, extraction methods, and processing technologies (Figure 3). Several studies reported that the antioxidant activity varied among different onion cultivars or varieties (5, 129–132), probably related to their genetic background (133). In addition, organic cultivation practices (28, 134), sulfur bentonite-organic-based fertilizers (135), and mycorrhizal fungi (136) were reported to improve the content of bioactive compounds with antioxidant activities in onions. The content of phenolics in the onion bulb and its antioxidant property were increased with the application of mycorrhizal inocula, humic acids, and elevated atmospheric CO_2_ (137). Planting time and density were also found to influence the antioxidant components of onion seeds (138). Besides, stored atmosphere conditions could affect the quality and bioaccessibility of total phenolics and antioxidant activity of the floral stem of the second-year onion resprout (139). Washing the fresh-cut onions with a combination of nisin and citric acid was reported to increase the total phenolic and flavonoid contents, and antioxidant capacity during storage (140). Sprouting of onion also increased its antioxidant activity, and contents of total phenolics and flavonoids (141, 142). Furthermore, the antioxidant activities in distinct parts of red onion, such as the dry skin and edible portion, were segregated based on the principal component analysis, probably due to the former rich in quercetin while the latter rich in quercetin-4-glucoside (35). The influences of food processing on antioxidant capacities of onions were investigated as well, including drying (143, 144), freezing (145), heating (41, 145), sautéing (38), and high-pressure processing (36). For instance, heating and freezing were found to reduce antioxidant activity of onion (145), while sautéing did not significantly change it (38).

Onion also exhibits antioxidant activity in cell and animal models. The expression of antioxidant enzymes, including catalase (CAT), NAD(P)H quinone dehydrogenase 1 (NQO1), and hemeoxygenase-1 (HO-1), was upregulated by onion extract in N27-A cells (6). In addition, several studies demonstrated that onion treatment could improve the antioxidant status of animals. Onion was effective for protection against oxidative stress by enhancing the activity of antioxidant enzymes, such as superoxide dismutase (SOD), CAT, and glutathione peroxidase (GPx), in hypercholesterolemic rats (10). The oxidative stress in the liver and kidney was ameliorated by pre-treatment with red onion peel extract in carbon tetrachloride-challenged rats (12). Onion fortified feed ameliorated the liver and kidney oxidative damages in rats administered with potassium bromate (54). Dietary addition of onion extract and combining onion peel powder with pawpaw seed were found to increase antioxidant enzyme activity in broiler chicks (55) and African catfish (56), respectively. Furthermore, a clinical trial revealed that drinking onion juice (100 mL) for 8 weeks could reduce total free radicals and superoxide anions levels, while elevate the glutathione content and total antioxidant capacity in healthy subjects (57).

Overall, onion exhibits strong antioxidant effects, and many factors could affect the antioxidant capability of onion. Although the antioxidant activity of onion has been extensively investigated, the related antioxidant molecular mechanism has been much less explored, which should be further clarified in the future.

### Antimicrobial Activity

Onion extracts and their derived bioactive compounds, such as thiosulfinate compounds, phenolic compounds, polysaccharides, and essential oils, have been reported to possess potent antibacterial properties (5, 30, 58, 146), antifungal activities (64, 66), and antiviral effects (147). Different drying methods, such as microwave drying, air drying, and freeze drying, were performed to evaluate the influence of drying processes on its antimicrobial activity, of which freeze-dried onion bulbs showed a stronger antimicrobial property (148).

Onion fiber-based composite materials combining with isolated flavonoids from onion skins were reported to exhibit certain antibacterial activity against *Staphylococcus aureus* and *Escherichia coli* (149). Moreover, it was demonstrated that gold nanoparticles (150), silver nanoparticles (151), graphene (60), and polymeric films (63) containing onion extracts exhibited excellent antibacterial properties against both gram-positive and gram-negative bacteria. Quorum sensing is very important for the coordination of bacterial virulence during infection. One result found that onion organic extracts and quercetin had interference on quorum sensing-regulated production of violacein and swarming motility in *Pseudomonas aeruginosa* and *Serratia marcescens*, of which quercetin aglycone reduced violacein production while quercetin aglycone and quercetin 3-β-D-glucoside inhibited bacterial motility (152). It is surprising that the biofilm formation, another crucial factor for antimicrobial resistance controlled by quorum sensing system, was not affected by the onion extracts or quercetin (152). In other studies, quorum sensing-mediated virulence factors in pathogens, such as biofilm formation, were found to be inhibited by the β-sitosterol derived compounds from onion husk extract (61) and quercetin 4′-*O*-β-D glucopyranoside from onion peel extract (153). Besides, onion essential oil was revealed to possess anti-biofilm activity against *Listeria monocytogenes* as well (154).

Onion essential oil was reported to showed fungicidal or inhibitory effects on the growth of fungal species isolated from food, including *Aspergillus, Fusarium*, and *Penicillium* species (64). Biosynthesis of silver nanoparticles using onion endophytic bacterium *Bacillus endophyticus* showed an effective antifungal effect against rice blast pathogen *Magnaporthe oryzae* with abnormal mycelia morphology and 88% inhibition rate of mycelium diameter (65). Besides, the ethanol extract of red onion was effective in preventing tinea pedis caused by the fungal infection of *Trichophyton rubrum* (66).

Therefore, onion has been demonstrated to inhibit the growth of microbes, showing great potential to be used as a natural preservative in the food industry, such as maintaining meat quality during refrigerated storage (155).

### Anti-inflammatory Activity

Onion also exhibited anti-inflammatory property, showing protective effects against inflammation-related diseases, such as neuroinflammation (6, 156), allergic inflammation (13), lung inflammation (68), colitis (14, 69), and paw edema (70). The anti-neuroinflammatory activities of onion extract were investigated in lipopolysaccharide (LPS)-induced BV-2 microglial cells. The methanol extract of onion could reduce the nitric oxide (NO) release by down-regulating the mRNA and protein levels of cyclooxygenase-2 (COX2) and inducible NO synthase (iNOS), and attenuate the elevation of proinflammatory cytokines, including tumor necrosis factor (TNF)-α, interleukin (IL)-1β, and IL-6 (6). Onion-derived nanoparticles and flavonoids from onion peels were reported to prevent the LPS-stimulated NO production in RAW264 cells (67) and BV-2 cells (156), respectively. Besides, the extract of onion bulb alleviated house dust mite-induced perivascular and peribronchial inflammation by inhibiting the epidermal growth factor receptor (EGFR)/extracellular signal-regulated kinase (ERK1/2)/protein kinase B (PKB/AKT) signaling pathway (13). Moreover, onion extract significantly reduced lung inflammatory cells, including monocyte, neutrophil, and eosinophil in asthmatic rats (68). Onion bulb extract was reported to both prevent and reverse colitis by regulating some pro-inflammatory signaling pathways, such as mechanistic target of rapamycin (mTOR), mitogen-activated protein kinase family (MAPK), cyclooxygenase-2 (COX-2), and tissue-inhibitors of metalloproteinases (TIMP), as well as several molecules involved in the apoptotic pathway, such as caspase-3, caspase-8, cytochrome c, B-cell lymphoma-extra-large (Bcl-XL), and Bcl-2 in mice (14, 69). In addition, intraperitoneally injection of onion aqueous extract dose dependently reduced Carrageenan-induced paw edema in rats (70). Therefore, onion can be a good food resource with anti-inflammatory activity for the prevention of inflammation-related diseases.

### Anti-obesity Activity

Promoting the browning of white adipose tissue is a promising strategy for the prevention of obesity. Quercetin from onion peel has been demonstrated to have browning effects in 3T3-L1 adipocytes and the white adipose tissue of mice (157). Several animal studies and clinical trials have reported that onion is effective in the prevention and management of obesity. Onion peel extract inhibited lipid accumulation in 3T3-L1 cells and reduced body weight in high-fat diet-fed mice *via* down-regulating the expression of lipogenesis-related genes (71). Red onion extract or quercetin supplementation could ameliorate obesity and insulin resistance in mice fed with a high-fat diet (72). Oral administration of onion oil prevented the body weight gain of rats triggered by high-fat diets (73). Moreover, dietary supplementation of onion peel extract was found to reduce the body weight, body fat mass, and percentage of body fat in overweight and obese Korean subjects (7, 76). Consumption of steamed onion resulted in a positive change of metabolic parameters, lowering the levels of triglycerides and C-peptide, and reduced the percentage of body fat, total body fat, visceral fat, and subcutaneous fat in overweight people (75). In addition, daily intake of onion powder improved the visceral fat area in healthy Japanese subjects with a high-density lipoprotein cholesterol level between 40 and 74 mg/dL (74). Generally, onion and its bioactive compounds have potential application in the management of obesity.

### Anti-diabetic Activity

**Figure 4:**
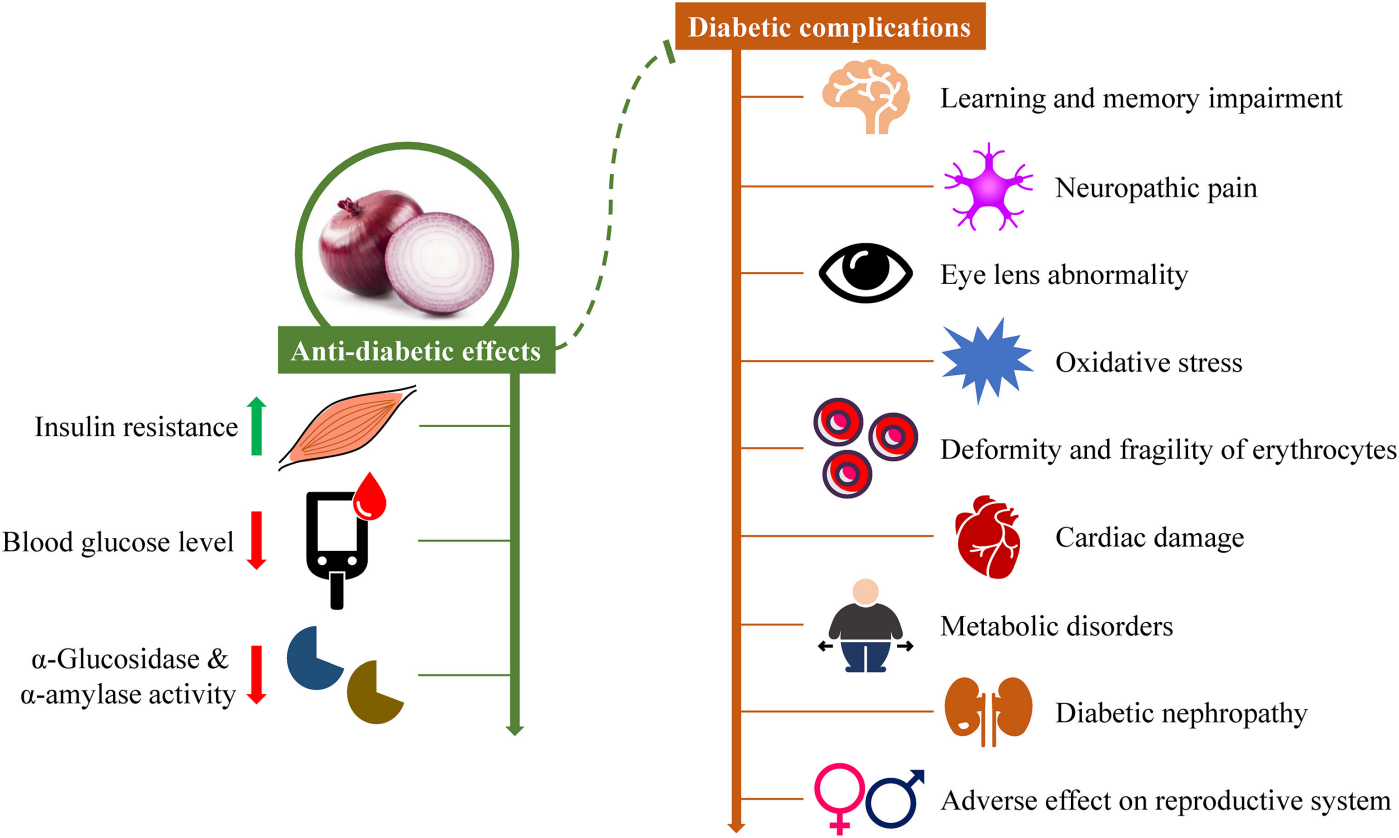
The effects of onion in diabetes and diabetic complications.

Increasing evidence from *in vitro* and *in vivo* studies have demonstrated that onions can not only ameliorate diabetes, but also treat different diabetic complications (Figure 4). Several studies indicate that onion exhibits antidiabetic potential *in vitro*. The extracts of onion skin or onion solid waste showed a remarkable inhibitory activity toward α-glucosidase and α-amylase, and the enzyme inhibitory activity was in a dose-dependent manner (158, 159). Besides, onion-based green synthesized silver nanoparticles were found to exhibit excellent α-glucosidase and α-amylase inhibitory activities (8). In another study, onion fiber concentrates were revealed to reduce starch digestibility and glucose production rate by suppressing α-amylase activity (160).

Onion also exhibits antidiabetic potential *in vivo*. The heat-processed onion extract was found to have a high content of Amadori rearrangement compounds, which reduced the post-prandial carbohydrate absorption and blood glucose levels by inhibiting intestinal sucrase activity in rats fed with sucrose or starch meals (85). Moreover, hyperglycemia and its associated metabolic disorders were reported to be ameliorated by dietary supplementation with fenugreek seeds and onion in diabetic rats (78). Hyperglycemia-induced osmotic and oxidative stress is a primary factor in the progression of diabetic complications. It has been reported that intake of fenugreek seeds and onion could also reduce oxidative stress (79), ameliorate eye lens abnormalities (82), alleviate cardiac damage (80), attenuate diabetic nephropathy (81, 86), and counter the deformity and fragility of erythrocytes (83) in STZ-induced diabetic rats. Tamtaji et al. (105) reported that the ethanolic extract of onion had a protective effect on learning and memory deficits in diabetic rats. Furthermore, the anti-diabetic activities of the inedible parts of onion, including skin, seeds, and leaves, have been investigated in STZ-induced diabetic rats as well. Red onion scales extract could improve the levels of fasting blood glucose and advanced glycation end products, enhance serum insulin level, and ameliorate diabetic nephropathy (84). The blood glucose and malondialdehyde (MDA) levels were declined in alloxan-induced diabetic rats fed with wheat bread supplemented with onion powder or onion peel extract (87). Onion seed extract showed a protective activity against the adverse side effects of diabetes in rats (77). The leaf extract of onion was found to ameliorate diabetes-induced neuropathic pain (106).

Onion exhibits antidiabetic potential in humans. In a randomized placebo-controlled clinical trial, daily ingestion of fresh yellow onion in breast cancer patients receiving doxorubicin-based chemotherapy was found to ameliorate the hyperglycemia and insulin resistance (96).

Overall, onion can fight against diabetes by reducing oxidative stress, moderating hyperglycemia, improving insulin resistance, and ameliorating various histopathological changes.

### Anticancer Activity

Allium vegetables receive extensive concerns because of their beneficial effects against numerous diseases, especially in treating cancer and alleviating the side effects of current anticancer therapies, which are associated with their bioactive compounds, such as sulfur compounds, flavonoids, and saponins (161, 162). The consumption of Allium vegetables was found to be negatively associated with the risk of diverse cancers, including breast cancer (163), gastric cancer (164), colorectal cancer (165), and upper aerodigestive tract cancers (166).

Many studies have been carried out to evaluate the anticancer activities of the common onion (92, 163, 167–169), other onion varieties, such as *Allium cepa* L. var. *proliferurn* Regel and *Allium cepa* L. Aggregatum group (90, 170, 171), and other Allium species, such as garlic (*Allium sativum* L.) (163, 172), leek (*Allium ampeloprasum* L.) (26), chive (*Allium schoenoprasum* L.), Welsh onion (*Allium fistulosum* L.) (173), Chinese onion (*Allium chinense*) (174, 175), and wild edible onions (*Allium flavum* and *Allium carinatum*) (176). By *in silico* approach, it has been found that onion-derived quercetin and diosgenin may take a role in the prevention and treatment of cancer by targeting on axon guidance receptor, neuropilin-1 (167). Onion extract or its major bioactive compounds showed potent anticancer activities, including cytotoxic, antiproliferative, anti-migratory, and apoptosis-inducing activities, in different cancer cells, such as human cervical carcinoma cell line (HeLa), myeloma cancer cell line (P3U1), pancreatic cancer cell line (AsPC-1), larynx cancer cell line (HEP2), colon cancer cell lines (SW620 and HCT116), adenocarcinoma cell line (Caco-2), glioblastoma cell line (A1235), liver cancer cell line (HepG2), and breast cancer cell lines (MDA-MB-231 and MCF-7) (26, 128, 130–133, 139–141]. 8-C-(E-phenylethenyl)quercetin, a novel compound from onion/beef soup, could cause G(2) phase arrest and inhibit the proliferation of colon cancer cells, and induced autophagic cell death, but not apoptotic cell death, by activating extracellular signal-regulated kinase (ERK) (168). Onion A, a sulfur compound from onion, exhibited antitumor effects by inhibiting the activation of suppressing signal transducer and activator of transcription-3 (STAT3) in myeloid lineage cells, and impaired the development of subcutaneous tumor and lung metastasis in tumor-bearing mice (95). Spiraeoside, isolated from red onion skin, exhibited promising anti-cancer effect against HeLa cell, could promote apoptosis by activating the expression of caspase-3 and caspase-9 (94). Recently, it was found that the anticancer activities of onion extract were enhanced by encapsulating on nano chitosan in multiple cancer cell lines (94). Besides, a wild edible onion showed a synergistic anticancer effect with doxorubicin against human hepatoma (HepG2) and lung carcinoma (A549) cells, and could protect from doxorubicin-induced cytotoxicity in human normal fibroblasts (MRC-5) and *in vivo* zebrafish models (176). Moreover, the intake of fresh onion was reported to reduce fasting blood glucose and improve insulin sensitivity in doxorubicin-treated breast cancer patients (96). Therefore, onion is an excellent anticancer vegetable, but its anticancer effect should be further verified in more clinical trials.

### Cardiovascular Protection

Studies have revealed that onion can effectively improve lipid profile and inhibit platelet aggregation, attenuating the incidence of cardiovascular diseases. The methanol extract, quercetin, and quercetin glucosides from onion were found to inhibit collagen-induced platelet aggregation by using rat platelet-rich plasma (97).

Many studies have focused on the hypocholesterolemic effect of onion and its bioactive compounds in rodents fed with high-cholesterol or high-fat diets. Onion could effectively decrease the levels of total cholesterol, triglyceride, and low-density lipoprotein cholesterol in hyperlipidemic animals (98, 101, 177). Li et al. (101) reported that polyphenol-rich onion extract ameliorated hyperlipidemia with upregulation of low-density lipoprotein receptor (LDLR) and downregulation of 3-hydroxy-3-methylglutaryl (HMG)-CoA reductase (HMGCR) in the liver of Sprague-Dawley rats. Kang et al. (98) found that quercetin-rich onion peel extract resulted in a higher level of fecal cholesterol, and lower values of atherogenic index and cardiac risk factor in high-cholesterol diet-fed mice, with upregulation of LDLR and cholesterol 7-α-monooxygenase (CYP7A1), indicating the cholesterol-lowering effect of onion *via* fecal excretion. The fecal bile acid content was reported to be modified by the dietary addition of onion in high-cholesterol diet-fed rats (178). Dietary onion significantly increased the activity of antioxidant enzymes and improved the anti-inflammatory response and cardiovascular risk biomarkers in rats fed with a high-cholesterol diet (10). Besides, the high-cholesterol diet-induced shifts in the lipid mediators, such as oxylipin and sphingolipid profiles, were also found to be modified by the dietary supplementation of onion in hypercholesterolemic Wistar rats (100). The antihyperlipidemic potentials of fermented onion and onion volatile oils were investigated as well, and both of them had certain positive effects on hyperlipidemia animals (73, 179). Moreover, the serum triglycerides levels were found to be reduced by consumption of steamed onion in overweight subjects (75). The intake of red wine extract of onion effectively reduced the total cholesterol and low-density lipoprotein cholesterol in healthy hypercholesterolemic subjects, while ameliorated the inflammatory responses and antioxidant defenses as well (102).

In addition, dietary fenugreek seeds and onion were reported to protect hyperglycemia-induced cardiac damage by inhibiting the activation of angiotensin-converting enzyme and angiotensin type 1 receptor in the heart of STZ-induced diabetic rats (80). Hypertension is known to be a key risk factor for cardiovascular disorders, however, the intake of quercetin from onion skin appeared to have no beneficial effects on blood pressure and endothelial function in subjects with hypertension (103, 104). More animal studies and clinical trials are needed for better understanding the cardiovascular protective effects and related mechanisms of onions and their bioactive compounds.

### Neuroprotection

Several studies have revealed that onion possesses anti-neuroinflammatory activity (6, 156), ameliorates neuropathic pain (106), and exerts neuroprotective effects against Parkinson's disease (11), memory impairment (105), cerebral injury (108), and retinal damage (107).

The anti-neuroinflammatory activity of onion and its bioactive compounds was investigated by using the LPS-stimulated BV2 microglia culture model (6, 156). Onion treatment prevented the LPS-induced increases of NO, TNF-α, IL-6, and IL-1β. The ameliorative effect of onion leave extract on neuropathic pain in rats was demonstrated by using two models, chronic constriction injury model and STZ-induced diabetic model (106). Onion leave extract significantly improved the behavioral and oxidative stress parameters as well as the sciatic nerve histopathological changes in both models. Onion ethanolic extract was reported to reduce malondialdehyde levels, ameliorate cognitive dysfunction, and prevent neuronal injury in 6-hydroxydopamine-induced rat model of Parkinson's disease (11). The neuroprotective effect of onion on learning and memory abilities was assessed in STZ-induced diabetic rats, and it was found that red onion ethanolic extract treatment could improve the learning and memory impairments in diabetic rats with reduced escape latency and traveled distance in Morris water maze test and increased step-through latency in passive avoidance test (105). The neuroprotective effect of onion on cerebral injury was evaluated in a cerebral ischemia/reperfusion mouse model, which was established by bilateral common carotid artery occlusion followed by reperfusion (108). It was revealed that the outer scale extract of onion could improve the memory and sensorimotor functions in cerebral injury mice by reducing cerebral infarct size and oxidative stress. The ischemia/reperfusion-induced retinal injury by pterygopalatine artery ligation in mice was used to investigate the neuroprotective effect of onion on neuronal damage, and it was found that onion water extract may protect from the retinal damage by regulating the expression of neurotrophic factors, such as B-cell lymphoma 2 (BCl-2), glial cell-derived neurotrophic factor (GDNF), glial fibrillary acidic protein (GFAP), and brain-specific homeobox/POU domain protein 3B (Brn3b) (107). Dietary fenugreek seeds and onion showed a protective effect on diabetic-cataract in STZ-induced diabetic rats (82).

### Hepatorenal Protection

The intake of functional natural products or constituents has been considered as a complementary way for the management of liver and kidney diseases. Onion is rich in multiple bioactive compounds with hepatorenal protective activities.

The phenolic-rich extract of red onion peels protected against carbon tetrachloride-induced oxidative stress in the liver and kidney tissues of rats (12). Dietary onion ameliorated antioxidant defense in hypercholesterolemic rats with increased activities of SOD, CAT, and GPx in the liver (10). The doxorubicin-mediated hepatotoxicity, parenchymal necrosis, and biliary duct proliferation, were alleviated by pre-treating with onion extract in rats and this hepatoprotective effect was attributed to the antioxidant capabilities of onion extract, which reduced the levels of glutathione and malondialdehyde, while enhanced the levels of SOD and GPx in the liver (109). Diabetic nephropathy, including renal architecture and functional abnormalities as well as podocyte damages, was attenuated by dietary fenugreek seeds and onion in rats by inhibiting the renin-angiotensin system and glucose transporters (86). The STZ-induced diabetic nephropathy in rats was also ameliorated by treating with red onion scales extract, and it was found to be associated with its metabolite fingerprint (84). Besides, several metabolites and related metabolic pathways in the liver were reported to be modulated by the onion supplementation in hypercholesterolemic Wistar rats (178). The liver weight, lipid profile, and lipid mediators were found to be ameliorated by onion in hypercholesterolemic animals (98, 100). Dietary intake of onion was revealed to lower hepatic steatosis, inflammation, and hepatic TNF-α expression in high-fat, high-sugar diet-fed rats (110). Moreover, the levels of alanine aminotransferase (ALT), a liver function marker, were significantly lower in healthy subjects with a daily intake of quercetin-rich onion, indicating that onion may be beneficial for improving liver function in humans (74).

### Respiratory System Protection

Onion has been recommended for the treatment of respiratory disorders, such as coughs, asthma, and bronchitis. Several studies have demonstrated that onion could relax tracheal smooth muscle (111), ameliorate allergic asthma (13), reduce lung inflammation (68), and attenuate lung damage (112).

Onion exhibited relatively potent relaxant effects on potassium chloride or methacholine-contracted tracheal smooth muscle in a dose-dependent manner, and the calcium channel blockade and/or β2-adrenergic stimulatory were involved in these effects (111). The onion bulb extract was reported to inhibit house dust mite-induced increase in airway cellular influx and goblet cell hyper/metaplasia, reduce *ex vivo* eosinophil chemotaxis, and ameliorate peribronchial and perivascular inflammation (13). The preventive and anti-inflammatory activities of onion on tracheal responsiveness and lung inflammation was demonstrated in asthmatic rats, and it was found that drinking water with onion extracts significantly reduced tracheal responsiveness and lung inflammatory cells, including monocytes, neutrophils, and eosinophils (68). Besides, the levels of antioxidant enzymes, SOD and CAT, were increased in bronchoalveolar lavage fluids of the ovalbumin-sensitized rats treated with onion extract, while the levels of oxidant markers, such as malondialdehyde, nitrogen dioxide (NO_2_), and nitrate (NO^3−^), were reduced (53). Reduction of lung malondialdehyde and elevation of lung SOD, CAT, and glutathione were also found in nicotine-induced lung damage rats treated with onion extract, which might be the main mechanism for the protective effect on lung damage (112). Moreover, the onion-derived bioactive compound, onionin A, was reported to inhibit lung metastasis in tumor-bearing mice (95).

Since the end of 2019, a new virus, the severe acute respiratory syndrome coronavirus 2 (SARS-CoV-2), has triggered the worldwide pandemic of coronavirus disease 2019 (COVID-19). Up to January 2021, data from the web-based COVID-19 dashboard has shown that more than 100 million people were infected with more than two million deaths (180). The infection of SARS-CoV-2 may result in severe pneumonia, and the common symptoms of COVID-19 include fever, cough, nasal congestion, sore throat, and breathing difficulties (181). Studies have demonstrated that onion and its bioactive compounds could attenuate lung inflammation and protect against diverse respiratory disorders. Therefore, it may be possible that onion and its bioactive compounds have the potential to fight against SARS-CoV-2 and can be consumed in our diets for the prevention of COVID-19, which still needs further investigation.

### Digestive System Protection

Onion has been shown to have a protective effect on the digestive system, such as mitigate gastric ulcer (113, 182), modulate gut microbiota (114–116, 183), and ameliorate colitis (14, 69, 116).

It has been reported that raw onion could inhibit histamine-induced gastric acid secretion and mitigate ethanol-stimulated gastric ulcer in rats, whereas boiled onion showed reduced potency (182). Alqasoumi (113) demonstrated that pre-treatment with onion could ameliorate gastric mucosal injury and ulcer index elicited by multiple factors, such as pylorus ligation, hypothermic restrainment, indomethacin, and necrotizing agents. Dietary supplementation of onion powders could modulate gut microbiota with an increased number of lactic acid bacteria in common carp juveniles (183). Onion could also be added into poultry feed as a natural growth promoter, which exhibited a positive effect on gut microbiota and intestinal histomorphology (55, 114). Onion-derived bioactive compounds, such as quercetin and quercetin monoglycosides, were found to enhance the enzymatic activity of gut microbiota in rats (115). Quercetin monoglycosides were reported to modulate the diversity of gut microbiota in colitis mice induced by dextran sodium sulfate (116). Moreover, the severity of colitis in mice was revealed to be reduced by administering with onion bulb extract, no matter it was given before, after, or at the same time of the colitis induction (14, 69). Besides, Allium species, including onion, showed protective effects against upper aerodigestive tract and gastrointestinal cancers (164–166, 168).

### Reproductive System Protection

Infertility has been considered a public health problem. Studies have shown that onion exhibited protective effects against adverse effects of chemical toxicity (119–122) and bacterial infection (118) on reproductive system, and could improve fertility and the quality of sperm and egg (15, 126).

Testosterone, a steroid hormone, plays a key role in the development of male sexual characteristics, and its reduction may cause infertility (184). It has been reported that onion extract-contained cysteine sulfoxides could stimulate the production of the testosterone precursor, progesterone, by activating the protein kinase A signaling pathway in a testis-derived cell line (I-10) (117). Bacterial infection is one of the main factors that cause infertility in men. The adverse effects of *Escherichia coli* infection were reduced in male rats receiving onion juice, while the total antioxidant capacity and testosterone level was increased (118). Besides, onion could restore the permethrin-induced reductions in hormonal levels of follicle-stimulating hormone (FSH) and luteinizing hormone (LH) as well as the expression of some key genes, such as luteinizing hormone/choriogonadotropin receptor (LHCGR) and steroidogenic factor 1 (SF1) (121). Onion also showed protective effects on testicle parameters and spermatogenesis in rats against the destructive effects of some insecticides or herbicides, such as permethrin, pyrethroid and paraquat (119, 120, 122). Onion seed extract was reported to have a protective activity against the negative effects of diabetes on the reproductive system in STZ-induced diabetic rats (77). Furthermore, the sperm quality and fertility were found to be improved in onion juice-treated adult male Wistar rats after the testicular torsion/detorsion (15). Recent study showed that onion juice could restore the erectile dysfunction induced by dutasteride in rats (125). Onion extract could prevent testicular damage induced by dexamethasone in rats (123) and the reproductive dysfunction in female rat offspring induced by maternal dexamethasone during lactation also found to be ameliorated by onion juice treatment (124). Dietary supplementation of onion extract was revealed to improve the egg quality and productive performance in laying hens (126). Moreover, onionin A showed an inhibitory effect on the progression of ovarian cancer by inhibiting tumor cell proliferation (9). In addition, results from clinical trials indicated that red onion intervention has certain beneficial effects in female subjects with polycystic ovarian syndrome (88, 185).

### Immune Modulation

Onion is thought to be beneficial for the immune system. Onion bulb extract showed an *in vitro* ability to induce the proliferation of human immune cells, particularly the CD^16+^ natural killer cells (127). Another study demonstrated that onion-derived onionin A could improve the activity of lymphocyte and prevent the immunosuppressive activities of macrophages and myeloid-derived suppressor cell in the tumor microenvironment (95). An *in vivo* study showed that onion extract had a protective effect against dexamethasone-induced immunosuppressive effects in Wistar rats, such as ameliorating white blood cell counts, enhancing antioxidant activities, and increasing the levels of cytokines (TNF and IL-6) and immunoglobulins (IgG and IgM) (16). The immunomodulatory property of onion was demonstrated in ovalbumin-sensitized rats, and lower levels of IL-4 and IgE were found in sensitized rats treated with onion extract (53). Moreover, onion could be used as a natural immunostimulant added to animal feed to improve the growth performance and reduce the occurrence of diseases (55, 183, 186).

### Other Health Functions of Onion

Browning of agro-products can lead to the deterioration of product quality and nutritional value, resulting in a decrease in consumer acceptance. Onion has been demonstrated to possess an inhibitory activity against the enzymatic browning reaction in fresh foods, such as fruit juice, mushrooms, and potato slices (187–190). In addition, intake of onion juice was reported to exhibit a beneficial effect on bone loss and bone mineral density by improving antioxidant capacity (57). Moreover, it has been revealed that fermented onion possessed an anti-photoaging effect against UVB-irradiation, probably by downregulating the expression of tyrosinase in B16F10 melanoma cells and collagenase-1 in UVB-induced HaCaT keratinocyte cells (191).

## Potential Safety Concerns of Onion

Onion has been consumed as a vegetable and applied as an herbal medicine for a long history. Normally, onion and its bioactive compounds are quite safe for humans. However, several potential safety risks have raised concerns, for example, the residue of pesticides (17), bioaccumulation of heavy metals (18, 19), and contamination of pathogenic microorganisms (20, 21), which are discussed below.

### Pesticide Residue

Pesticides with low toxicity and rapid degradation can be recommended for use in crops. However, the improper use of some pesticides in the onion crop may still cause health risks from onion consumption. Several studies have been carried out to investigate the degradation behavior, residue distribution, and dietary risk of different pesticides, including insecticides (17, 192), fungicides (193–195), and herbicides (196), which were used for pest and disease protection and weed control in onion planting. Overall, the dietary risk of these pesticides through onion could be negligible with the reasonable usage does of pesticides and enough preharvest interval (Table 2).

**Table 2:** The residue decline and recommend preharvest interval of commonly used pesticides in onion.

**Pesticides**	**Applied dosage**	**Half-life**	**Preharvest interval**	**References**
Thiacloprid	48 g a.i./ha (1×)	1.92 days	9 days	(192)
	96 g a.i./ha (2×)	2 days		
Spinetoram	0.031 g a.i./ha (1×)	1.2 days	1 day	(17)
Spinosad	30 g a.i./ha (1×)	1.42 days	0 day	
Propiconazole	120 g a.i./ha (1×)	6.1–6.2 days		(194)
	180 g a.i./ha (1.5×)			
Tebuconazole	215 g a.i./ha (1×)	1.7 days	12 days	(195)
	430 g a.i./ha (2×)	2.1 days		
Fluopyram plus Tebuconazole	75 + 75 g a.i./ha (1×)	8.8 days	7 days	(193)
	150 + 150 g a.i./ha (2×)	9.1 days		

*a.i./ha, active ingredient per hectare; ×, recommended dosage*.

### Heavy Metal Enrichment

The enrichment of heavy metals, such as cadmium (Cd), lead (Pb), chromium (Cr), and nickel (Ni), in the farmland growing onion may induce the accumulation of heavy metals in onion and induce food safety issue. Bystricka et al. (197) reported that the content of Cd, Cr, and Pb exceeded the reference limits in the dry matter of onions from contaminated soil in the Slovak Republic, and different heavy metal enrichment capacities were found among the onion varieties. Cd and Pb contents in some Malaysian onions were reported beyond the permissible levels, and the planting site had a greater impact than onion varieties on heavy metal enrichment (19). Gashi et al. (198) demonstrated that the activity of delta-aminolevulinic acid dehydratase could be used as a sensitive biomarker for the risk assessment of bioaccumulation of heavy metals in onion. Yao et al. (199) developed a novel *in situ* imaging strategy for the fast evaluation of heavy metal enrichment in onion. Besides, Cd uptake in onion and other crops was revealed to be affected by environmental and edaphic factors (200). Indeed, the Cd accumulation in onion was reported to be mitigated by increasing the soil pH (18) or the application of silicon fertilizer to the soil (201).

### Microbial Contamination

The use of sanitizer, sodium hypochlorite, combined with elevated CO_2_/reduced O_2_ in package atmosphere was found beneficial for the safety and quality of fresh-cut onion, which showed effective inhibitory effects on the growth of *Salmonella typhimurium*, mesophilic aerobic bacteria, yeasts, and molds during storage (202). Moreover, the pathogenic microorganisms, including *Listeria monocytogenes* (21), *Escherichia coli* (203, 204), *Salmonella* spp. (205), and mold (20) in onion infected by contaminated irrigation water or transferred from contaminated workplace are also considered as potential health risks. The microwave-integrated cold plasma treatment was reported to be a potential technology for non-thermal decontamination of onion powder (206). In addition, improving monitoring of the quality of irrigation water as well as the regular decontamination of the workplace may be the effective ways to reduce the risk of pathogen exposure in onion.

## Conclusions

Onion is a widely cultivated and consumed vegetable, and contains various bioactive components. The sulfur-containing compounds, such as onionin A and cysteine sulfoxide, as well as the phenolic compounds, such as quercetin and quercetin glucosides, are the main bioactive constituents in onion and contribute to its multiple health functions, including antioxidant, antimicrobial, anti-inflammatory, and immunomodulatory properties. Moreover, onion can be a promising natural resource to develop functional foods or nutraceuticals for the prevention and management of certain diseases, such as obesity, diabetes, cancers, cardiovascular diseases, neurodegenerative diseases, nephropathy, respiratory disorders, colitis, and infertility.

At present, both *in vitro* and *in vivo* evidence suggests that onion powder, juice, and extracts exhibit multiple health functions. Although several bioactive compounds have been found to contribute to these functions, more bioactive compounds from onion or its by-products should be identified, and their health functions, the relevant molecular mechanisms, and whether they have synergistic effects need to be illuminated in the future. Currently, most studies focused on the health functions of the raw onion, it is necessary to investigate whether the cooking processing can impact its health benefits. Moreover, more well-designed clinical trials are still required to verify the health benefits of onion and onion-derived bioactive compounds in humans. Last but not the least, safety issues of onion should always be aware of, not only the contaminations mentioned in this review, but also other potential risks, such as the overdose of bioactive compounds.

## Author Contributions

X-XZ, F-JL, and HL drafted the manuscript. H-BL, D-TW, FG, WM, YW, B-HM, and R-YG critically revised the manuscript. R-YG and B-HM conceived the idea and scientific guidance through the process. All authors have read and agreed to the published version of the manuscript.

## Conflict of Interest

The authors declare that the research was conducted in the absence of any commercial or financial relationships that could be construed as a potential conflict of interest.
